# A new ovarian response prediction index (ORPI): implications for individualised controlled ovarian stimulation

**DOI:** 10.1186/1477-7827-10-94

**Published:** 2012-11-21

**Authors:** Joao Batista A Oliveira, Ricardo LR Baruffi, Claudia G Petersen, Ana L Mauri, Adriana M Nascimento, Laura Vagnini, Juliana Ricci, Mario Cavagna, Jose G Franco

**Affiliations:** 1 Centre for Human Reproduction Prof. Franco Junior, Preto, Ribeirao, Brazil; 2 Paulista Centre for Diagnosis, Research and Training, Preto, Ribeirao, Brazil; 3 Department of Gynaecology and Obstetrics, Botucatu Medical School, São Paulo State University, Botucatu, UNESP, Brazil; 4 Women’s Health Reference Centre, Perola Byington Hospital, Paulo, Sao, Brazil

**Keywords:** Ovarian response prediction index, Individualised controlled ovarian stimulation, Anti-Müllerian hormone, Antral follicles, Age

## Abstract

**Background:**

The objective was to present a new ovarian response prediction index (ORPI), which was based on anti-Müllerian hormone (AMH) levels, antral follicle count (AFC) and age, and to verify whether it could be a reliable predictor of the ovarian stimulation response.

**Methods:**

A total of 101 patients enrolled in the ICSI programme were included. The ORPI values were calculated by multiplying the AMH level (ng/ml) by the number of antral follicles (2–9 mm), and the result was divided by the age (years) of the patient (ORPI=(AMH x AFC)/Patient age).

**Results:**

The regression analysis demonstrated significant (*P*<0.0001) positive correlations between the ORPI and the total number of oocytes and of MII oocytes collected. The logistic regression revealed that the ORPI values were significantly associated with the likelihood of pregnancy (odds ratio (OR): 1.86; *P*=0.006) and collecting greater than or equal to 4 oocytes (OR: 49.25; *P*<0.0001), greater than or equal to 4 MII oocytes (OR: 6.26; *P*<0.0001) and greater than or equal to 15 oocytes (OR: 6.10; *P*<0.0001). Regarding the probability of collecting greater than or equal to 4 oocytes according to the ORPI value, the ROC curve showed an area under the curve (AUC) of 0.91 and an efficacy of 88% at a cut-off of 0.2. In relation to the probability of collecting greater than or equal to 4 MII oocytes according to the ORPI value, the ROC curve had an AUC of 0.84 and an efficacy of 81% at a cut-off of 0.3. The ROC curve for the probability of collecting greater than or equal to 15 oocytes resulted in an AUC of 0.89 and an efficacy of 82% at a cut-off of 0.9. Finally, regarding the probability of pregnancy occurrence according to the ORPI value, the ROC curve showed an AUC of 0.74 and an efficacy of 62% at a cut-off of 0.3.

**Conclusions:**

The ORPI exhibited an excellent ability to predict a low ovarian response and a good ability to predict a collection of greater than or equal to 4 MII oocytes, an excessive ovarian response and the occurrence of pregnancy in infertile women. The ORPI might be used to improve the cost-benefit ratio of ovarian stimulation regimens by guiding the selection of medications and by modulating the doses and regimens according to the actual needs of the patients.

## Background

For ovarian stimulation in in vitro fertilisation (IVF) cycles, different protocols have been developed to induce multifollicular development, which increases the number of available oocytes and, thereby, the number of embryos for selection and transfer [1]. However, the patients are exposed to the possibility of a low or excessive ovarian response. Furthermore, the possibility of a negative impact of supraphysiological levels of oestrogen resulting from the large numbers of follicles and oocytes on the embryo quality and/or the endometrium has been repeatedly questioned [2-4]. For this reason, knowledge of the patient’s potential ovarian response can help clinicians individualise the medication dosage, which may reduce the adverse effects of an excessive ovarian response, decrease the rate of cancelled cycles and ultimately, increase the pregnancy rate.

The first indicator of the ovarian reserve taken into account is the patient’s age. Although the number and quality of oocytes both decrease with age, the reproductive potential varies drastically among women of similar age; therefore, they might exhibit different responses to ovarian stimulation [5]. Consequently, an individual’s chronological age may not be as valuable a predictor of fertility as her “biological age”, as defined by hormonal and functional profiles [6]. In fact, in addition to age, several clinical, endocrine and ultrasound markers, and dynamic tests have been proposed for the prediction of the ovarian response to stimulation [7,8]. Among these markers, use of the level of anti-Müllerian hormone (AMH) and the antral follicle count (AFC) is of particular interest [7-16]. The AFC consists of the sum of follicles <10 mm in both ovaries on a transvaginal ultrasound and has been used to predict the ovarian reserve and the patient response to ovarian stimulation. However, there is significant variation among different authors in the limits used to classify antral follicles [7,12,13,17-20]. AMH, a member of the transforming growth factor-beta superfamily, is only produced by the granulosa cells surrounding the pre-antral and small antral follicles. Additionally, AMH is independent of follicle-stimulating hormone (FSH), whereby its levels are a direct measure of the follicular pool production. The serum levels of AMH decrease throughout reproductive life and are undetectable in the postmenopausal period [14].

However, despite the predictive power that each marker for the ovarian response may have individually, all of these markers have errors associated with their estimation. In fact, none of these parameters can be considered to be undoubtedly reliable predictors of the number/quality of the remaining oocytes in the ovary or the probability of pregnancy following infertility treatment [17,21,22]. A systematic review of tests predicting the ovarian reserve and IVF outcomes [7] observed that the accuracy of the so-called ovarian reserve tests in predicting the occurrence of both a poor ovarian response and hyperstimulation appears to be modest. Therefore, a prediction of the ovarian response using a single biomarker may not be sufficient for the formulation of a precise treatment plan.

Considering these observations, the main objectives of the present study were to present a new ovarian response prediction index (ORPI), which was based on the AMH, AFC and age, and to verify whether such a marker could be a reliable predictor of ovarian stimulation in assisted reproductive technology (ART) cycles.

## Methods

### Patients

This study included 101 patients attending their first ICSI (intracytoplasmic sperm injection) cycle at the Centre for Human Reproduction “Prof. Franco Junior” between March 2012 and August 2012. All patients satisfied the following criteria: age ≤39 years, body mass index (BMI) between 20–30 kg/m^2^, regular menstrual cycles, both ovaries present, no history of ovarian surgery, no severe endometriosis and no evidence of endocrine disorders. The only exclusion criterion was the presence of ovarian cysts as assessed by transvaginal ultrasound. The study was authorised by the local ethical committee Institutional Review Board, and a written informed consent was obtained from all recruited subjects.

### AMH measurement

A venous blood sample for an AMH measurement was taken before the scheduled treatment (minimum of 30 days) during the early follicular menstrual cycle phase in all women. AMH was measured using an enzymatically amplified 2-site immunoassay kit (AMH Gen II ELISA, Beckman Coulter Inc.) according to the manufacturer’s manual. The lowest detection limit of this assay is 0.01 ng/ml, whereas the maximum intra- and inter-assay coefficients of variation are 3.3% and 6.5%, respectively. To minimise the chances of bias in the assay, all sera were processed in duplicate during the same day, using the same measurement kits, and by the same operator. Low- and high-level controls were included in each assay.

### Antral follicles count

All subjects had a transvaginal ultrasonographic evaluation performed during the early follicular phase of a previous cycle before the scheduled treatment. A single experienced sonographer, who was blinded to the results of any hormonal assays and the patient’s age, performed the evaluation using a conventional 2-dimensional transvaginal ultrasound at 7 MHz (Medison Digital Color MT; Medison Co. Ltd., Seoul, Korea). The total number of 2-9-mm antral follicles in both ovaries was used for the calculations. The intra-observer coefficient of variation was 1.0%.

### Ovarian stimulation protocol

The patients were subjected to 2 schemes of controlled ovarian stimulation, as follows: a long gonadotropin-releasing hormone (GnRH) agonist (GnRH-a; n=60) protocol or a multi-dose GnRH antagonist (GnRH-ant/n=41) protocol. The selection of the stimulation protocol was at the discretion of the clinician.

*GnRH-a protocol*[23-27]: The pituitary downregulation began during the luteal phase of the previous menstrual cycle with the GnRH-a leuprolide acetate (leuprolide acetate; Lupron®; Abbott, Brazil) at a dose of 1 mg/day for 14 days. The ovaries were then stimulated with a fixed dose of 150–225 IU of recombinant FSH (rFSH; Gonal F®; Serono, Brazil) and 75 IU/day of recombinant luteinising hormone (rLH; Luveris®; Serono, Brazil) for a period of 7 days. The decision on the starting dose of FSH was based on patient’s age. On day 8 of the ovarian stimulation, the follicular development was monitored by a transvaginal ultrasound at 7 MHz. The rFSH dose was modified according to the ovarian response, and the rLH supplementation was increased to 150 IU/day when one or more follicles measuring ≥10 mm in diameter were found.

*GnRH-ant protocol*[23-27]: On day 3 of the cycle, ovarian stimulation was induced with a fixed dose of 150–225 IU of rFSH and 75 IU/day of rLH for a period of 5 days. The decision on the starting dose of FSH was based on patient’s age. On day 8 of the menstrual cycle (day 6 of ovarian stimulation), the follicular development was monitored by a transvaginal ultrasound at 7 MHz. The r-FSH dose was modified according to the ovarian response, and the r-LH supplementation was increased to 150 IU/day when 1 or more follicles measuring ≥10 mm in diameter were found. The GnRH-ant cetrorelix (cetrorelix; Cetrotide®; Serono, Brazil) was started at a dose of 0.25 mg/day s.c. when at least 1 follicle of ≥14 mm was observed by the ultrasound.

To induce the final oocyte maturation in both protocols (GnRH-a and GnRH-ant), 250 μg of recombinant human chorionic gonadotropin (r-hCG; Ovidrel; Serono, Brazil) was administered s.c. when at least 2 follicles reached a mean diameter of ≥17 mm. GnRH-a and GnRH-ant were administered until the day of the r-hCG injection. The oocyte retrieval was performed by a transvaginal aspiration under ultrasound guidance 34–36 hours following the r-hCG injection.

### Calculation of ovarian response prediction index (ORPI)

The ORPI values were calculated by multiplying the AMH (ng/ml) level by the number of antral follicles (2–9 mm), and the result was divided by the age (years) of the patient. This definition of ORPI was based on previous evaluations that found that the ovarian response to stimulation had positive correlations with the AMH levels and number of antral follicles and was negatively correlated with the patient’s age. The derivation of ORPI was intuitive, based on the observed correlations and testing of different combinations. We sought a simple index that was easy to use in daily practice and combined a small number of variables whose association could potentiate the result of each individual variable in predicting ovarian response to stimulation and at the same time compensate for possible individual deficiencies. The ORPI was defined by the following equation:ORPI=(AMH x AFC)/Patient age.

Notably, the calculated value of the ORPI in the study was not influenced by the protocol choice for the induction of ovulation or the doses of gonadotropin.

### Endpoints

The primary endpoints were the total number of oocytes and the number of metaphase II (MII) oocytes retrieved. The secondary endpoints were the number of follicles ≥10 mm, ≥16 mm and ≥18 mm on the day of HCG administration and clinical pregnancy.

### Statistical analysis

The data management and univariate analysis were performed using the StatsDirect statistical software (Cheshire UK). The values for the ORPI, age, AMH, AFC, total number of oocytes retrieved, number of MII oocytes and the number of follicles ≥10 mm, ≥16 mm and ≥18 mm on the day of hCG administration were treated as continuous variables for analysis.

The Mann–Whitney test, Student’s *t*-test and the chi-square test were used when appropriate. Correlations were performed using the Spearman’s rank correlation test. A *P*<0.05 was considered statistically significant. A univariate logistic regression was used to estimate the value of an independent variable in predicting the likelihood of collecting ≥4 oocytes (criterion for the classification as a poor ovarian response) [22,28-30], collecting ≥4 MII oocytes, collecting ≥15 oocytes (assessing excessive response) [19,31-35] and clinical pregnancy (determined based on the presence of a gestational sac accompanied by an image of the embryonic/foetal cardiac activity on transvaginal ultrasounds 4 weeks after transfer). The odds ratio (OR) and 95% confidence interval (CI) constituted the descriptive analysis.

Receiver operating characteristic (ROC) curves were constructed to examine the performance of the ORPI in predicting clinical pregnancy and the retrieval of ≥4 oocytes, ≥4 MII oocytes and ≥15 oocytes. An optimised threshold was determined. The discriminative performance of the model was assessed by the area under the curve (AUC) of the ROC curve. Sensitivity was defined as the fraction of cycles in which the expected outcome (clinical pregnancy and retrieval of ≥4 oocytes, ≥4 MII oocytes and ≥15 oocytes) was predicted correctly, and the specificity was defined as the fraction of cycles not resulting in the expected outcome that was predicted correctly. StatsDirect requires the following 2 columns of data for each ROC plot: a column with the test results for cases where the condition being tested is known to be present and another column with the test results for known negative cases. The sensitivity is then plotted against specificity. StatsDirect calculates the area under the ROC curve directly using an extended trapezoidal rule and by a non-parametric method that is analogous to the Wilcoxon/Mann–Whitney test. A confidence interval was constructed using DeLong’s variance estimate.

## Results

The general characteristics of the study population are summarised in Table 1. Of all 101 women, the mean age was 34.1±5.1 years (range 21–39), the mean AMH level was 1.8±1.8 ng/mL (range 0.01-9.6) and the mean AFC was 12.5±6.2 (range 2–34). The mean ORPI was 1.0±1.5 (range 0–8.8). Basic demographic characteristics such as maternal age, BMI, duration of infertility, smoking, alcohol use and infertility aetiology were not significantly different (*P*>0.05) between the GnRH-a and GnRH-ant patient groups. The distribution (*P*>0.05) of the main characteristics of the ovarian stimulation cycle observed for the GnRH-a and GnRH-ant groups were comparable.

**Table 1:** General characteristics of the study population

	**General population (n=101)**	**GnRH agonist protocol (n=60)**	**GnRH antagonist protocol (n=41)**	***P***
*Age (years)*	34.1±5.1 (21–39)	34.1±5.4 (21–39)	34.1±4.7 (26–39)	ns
*AMH (ng/ml)*	1.8±1.8(0.01-9.6)	1.5±1.3 (0.01-8.2)	2.3±2.2 (0.01-9.6)	ns
*AFC (n) (2–9 mm)*	12.5±6.2 (2–34)	11.8±5.3 (2–34)	13.6±7.1 (4–28)	ns
*ORPI*	1.0±1.5(0–8.8)	0.7±1.2(0–8.8)	1.3±1.8(0–7.6)	ns
*BMI*	24.6±4.1	24.0±4.1	25.6±3.8	ns
*Tobacco use*	4% (4/101)	3.3% (2/60)	4.9% (2/41)	ns
*Regular alcohol use*	1% (1/101)	1.7% (1/60)	0/41	ns
*Time of infertility (years)*	4.9±3.3	5.1±3.6	4.1±2.7	ns
*Aetiology (%)*				ns
*-Male*	35.6% (36/101)	41.6% (25/60)	26.8% (11/41)	
*-Idiopathic*	25.7% (26/101)	20% (12/60)	34.1% (14/41)	
*-Tuboperitoneal*	17.8% (18/101)	16.7% (10/60)	19.5% (8/41)	
*-Endometriosis*	12.9% (13/101)	15% (9/60)	9.8% (4/41)	
*-Tuboperitoneal+endometriosis*	4% (4/101)	3.3% (2/60)	4.9% (2/41)	
*-Male+endometriosis*	3% (3/101)	1.7% (1/60)	4.9% (2/41)	
*-Male+tuboperitoneal*	1% (1/101)	1.7% (1/60)	0.0% (0/41)	
*Infertility*				ns
*-Primary*	78.2% (79/101)	81.7% (49/60)	73.2% (30/41)	
*-Secondary*	21.8% (22/101)	18.3% (11/60)	26.8% (11/41)	
*Total dose FSH (UI)*	1956±804	2136±774	1692±782	ns
*Total dose LH (UI)*	982±351	1035±322	903±380	ns
*Time of stimulation (days)*	10.2±2.1	10.6±2.1	9.5±1.9	ns
*Follicles (n) (hCG day)*				
*-≥10 mm*	12.8±8.6	12.8±7.3	12.8±10.4	ns
*-≥16 mm*	5.7±3.5	6.2±3.4	4.9±3.5	ns
*-≥18 mm*	3.8±2.6	4.2±2.2	3.3±2.9	ns
*Retrieved oocytes*				
*-Total*	9.7±7.1	9.7±6.0	9.6±8.4	ns
*-Metaphase II stage*	6.9±5.2	7.1±4.6	6.6±6.0	ns
*-Metaphase I stage*	1.3±1.8	1.2±1.6	1.5±2.1	ns
*-Germinal vesicle stage*	0.8±1.1	0.8±1.2	0.7±1.0	ns
*Clinical pregnancy rate*	32.7% (33/101)	31.2% (19/60)	34.1% (14/41)	ns

AMH: anti-Müllerian hormone ORPI: ovarian response prediction index.

AFC: antral follicle count BMI: body mass index.

ns: not significant.

The regression analysis demonstrated significant (*P*<0.05) positive correlations between the ORPI and the total number of oocytes collected (*r*=0.78), total number of MII oocytes (*r*=0.70) and the number of follicles ≥10 mm (*r*=0.82), follicles ≥16 mm (*r*=0.67) and follicles ≥18 mm (*r*=0.56) on the hCG administration day. Additionally, all the other markers of ovarian response showed statistically significant correlations with the variables analysed. However, the association provided by the ORPI improved the correlation because the individual correlation coefficients of each marker of ovarian response (age, AMH and AFC) were always lower than that presented by the ORPI. When the analysis was performed considering only the GnRH-a protocol group, the regression analysis also demonstrated significant (*P*<0.05) positive correlations between the ORPI and the total number of oocytes collected, total number of MII oocytes and the number of follicles ≥10 mm, ≥16 mm and ≥18 mm. Similarly, the analysis considering only the GnRH-ant protocol group also showed statistically significant (*P*<0.05) correlations between the ORPI with all variables analysed. Table 2 summarises these results.

**Table 2:** Correlation between predictors of the ovarian response (age, AMH, AFC and ORPI) and the total number of oocytes collected, total number of MII oocytes collected and the number of follicles ≥10 mm, ≥16 mm and ≥18 mm at the time of hCG administration

**Ovarian response markers**	**Variable Analyzed**								
	**Total oocytes collected**								
	**General population**			**GnRH agonist group**			**GnRH antagonist group**		
	r	95% CI	*P*	r	95% CI	*P*	r	95% CI	P
**Age**	−0.49	−0.63 to −0.32	<0.0001	−0.44	−0.62 to −0.20	0.0004	−0.57	−0.75 to −0.31	<0.0001
**AMH**	0.72	0.60 to 0.80	<0.0001	0.74	0.60 to 0.84	<0.0001	0.70	0.50 to 0.83	<0.0001
**AFC**	0.72	0.61 to 0.81	<0.0001	0.67	0.50 to 0.79	<0.0001	0.80	0.66 to 0.89	<0.0001
**ORPI**	0.78	0.68 to 0.84	<0.0001	0.78	0.66 to 0.87	<0.0001	0.81	0.65 to 0.89	<0.0001
	**MII oocytes**								
	**General population**			**GnRH agonist group**			**GnRH antagonist group**		
	r	95% CI	*P*	r	95% CI	*P*	r	95% CI	*P*
**Age**	−0.51	−0.64 to −0.34	<0.0001	−0.47	−0.65 to −0.24	0.0001	−0.58	−0.75 to −0.32	<0.0001
**AMH**	0.64	0.50 to 0.74	<0.0001	0.69	0.52 to 0.80	<0.0001	0.61	0.37 to 0.78	<0.0001
**AFC**	0.64	0.50 to 0.74	<0.0001	0.59	0.40 to 0.74	<0.0001	0.69	0.51 to 0.84	<0.0001
**ORPI**	0.70	0.57 to 0.78	<0.0001	0.74	0.59 to 0.83	<0.0001	0.70	0.48 to 0.83	<0.0001
	**Follicles ≥10 mm**								
	**General population**			**GnRH agonist group**			**GnRH antagonist group**		
	r	95% CI	*P*	r	95% CI	*P*	r	95% CI	*P*
**Age**	−0.50	−0.64 to −0.33	<0.0001	−0.44	−0.62 to −0.20	0.0005	−0.60	−0.77 to −0.35	<0.0001
**AMH**	0.77	0.67 to 0.84	<0.0001	0.80	0.68 to 0.88	<0.0001	0.77	0.61 to 0.88	<0.0001
**AFC**	0.76	0.65 to 0.83	<0.0001	0.76	0.62 to 0.85	<0.0001	0.79	0.63 to 0.88	<0.0001
**ORPI**	0.82	0.74 to 0.87	<0.0001	0.86	0.76 to 0.91	<0.0001	0.81	0.72 to 0.91	<0.0001
	**Follicles ≥16 mm**								
	**General population**			**GnRH agonist group**			**GnRH antagonist group**		
	r	95% CI	*P*	r	95% CI	*P*	r	95% CI	*P*
**Age**	−0.51	−0.65 to −0.35	<0.0001	−0.46	−0.64 to −0.23	0.0002	−0.62	−0.78 to −0.38	<0.0001
**AMH**	0.60	0.45 to 0.71	<0.0001	0.65	0.47 to 0.78	<0.0001	0.58	0.33 to 0.76	<0.0001
**AFC**	0.61	0.46 to 0.72	<0.0001	0.64	0.45 to 0.77	<0.0001	0.61	0.42 to 0.80	<0.0001
**ORPI**	0.67	0.53 to 0.76	<0.0001	0.72	0.56 to 0.82	<0.0001	0.67	0.45 to 0.81	<0.0001
	**Follicles ≥18 mm**								
	**General population**			**GnRH agonist group**			**GnRH antagonist group**		
	r	95% CI	*P*	r	95% CI	*P*	r	95% CI	*P*
**Age**	−0.44	−0.59 to −0.26	<0.0001	−0.35	−0.56 to −0.10	0.0053	−0.66	−0.81 to −0.44	<0.0001
**AMH**	0.50	0.33 to 0.63	<0.0001	0.50	0.31 to 0.69	<0.0001	0.56	0.30 to 0.74	0.0001
**AFC**	0.52	0.35 to 0.65	<0.0001	0.52	0.32 to 0.70	<0.0001	0.62	0.38 to 0.78	<0.0001
**ORPI**	0.56	0.40 to 0.68	<0.0001	0.56	0.37 to 0.72	<0.0001	0.65	0.42 to 0.80	<0.0001

AMH: anti-Mullerian hormone; ORPI: ovarian response prediction index; AFC: antral follicle count;

CI: confidence interval.

**Figure 1:**
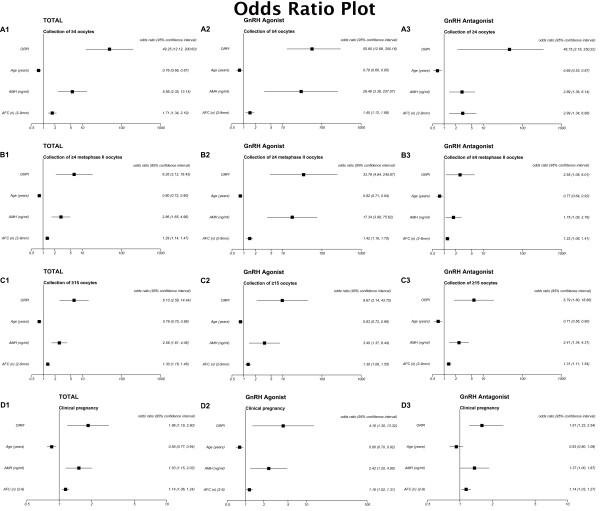
**Logistic regression analysis for the prognostic factors regarding the collected oocytes and pregnancy occurrence. A**. Collection of ≥4 oocytes. **B**. Collection of ≥4 MII oocytes. **C**. Collection of ≥15 oocytes. **D**. Clinical pregnancy.

The logistic regression analysis revealed that the ORPI values were significantly associated with the likelihood of clinical pregnancy (OR: 1.86; *P*=0.006) and of collecting ≥4 oocytes (OR: 49.25; *P*<0.0001), ≥4 metaphase II oocytes (OR: 6.26; *P*<0.0001) and ≥15 oocytes (OR: 6.10; *P*<0.0001). Alternatively, the logistic regression analysis also revealed a statistically significant (*P*<0.05) association between the number and maturity of collected oocytes and the other prognostic factors analysed, including the woman’s age (≥4 oocytes: OR: 0.76; ≥4 MII oocytes: OR: 0.80; ≥15 oocytes: OR: 0.79; clinical pregnancy: OR: 0.85), AMH (≥4 oocytes: OR: 5.56; ≥4 MII oocytes: OR: 2.86; ≥15 oocytes: OR: 2.56; clinical pregnancy: OR: 1.53) and AFC (≥4 oocytes: OR: 1.71; ≥4 MII oocytes: OR: 1.29; ≥15 oocytes: OR: 1.30; clinical pregnancy: OR: 1.14). However, the odds ratios presented by the ORPI were always higher (i.e., further from 1) than those presented by all other prognostic factors. The results indicate that for each one unit increase of the ORPI value, the chance of collecting ≥4 oocytes increases 49 times (or it increases by 4.9 times for each increase of 0.1), 6 times for collecting ≥4 MII oocytes ≥15 oocytes and 1.8 times for the occurrence of clinical pregnancy. These results indicate that the ORPI presents a predictive capability for the occurrence of these events (collection of ≥4 oocytes, ≥4 MII oocytes and ≥15 oocytes and pregnancy) that was higher than that of each marker individually. As shown by the regression analysis, the results obtained when considering patients undergoing GnRH-a protocol and GnRH-ant protocol individually replicated the pattern obtained for the total population (the odds ratios presented by the ORPI were always higher than those presented by all other prognostic factors). Figure 1 summarises these results.

**Figure 2:**
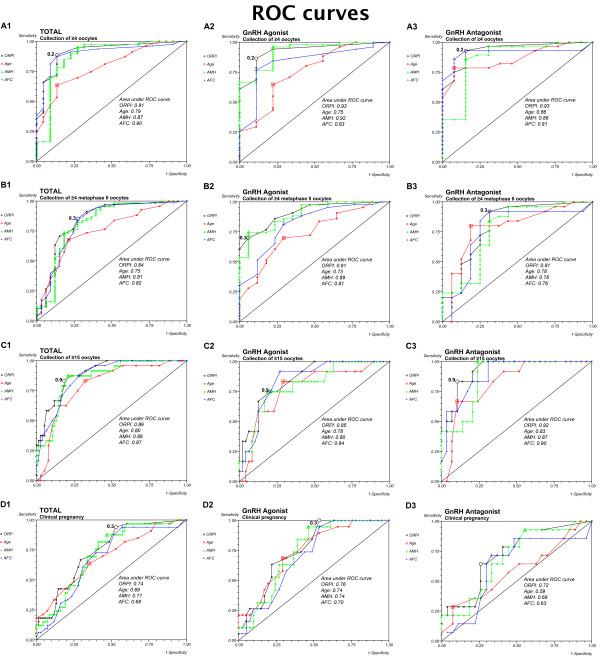
**ROC Curve**. The ROC curve analysis for ORPI as a prognostic factor regarding the collected oocytes and pregnancy occurrence. **A**. Collection of ≥4 oocytes. **B**. Collection of ≥4 metaphase II (MII) oocytes. **C**. Collection of ≥15 oocytes. **D**. Clinical pregnancy.

The performance of the ORPI as a prognostic test was observed using ROC curves. Regarding the probability of collecting ≥4 oocytes, the ROC curve showed an area under the curve of 0.91 (95% CI: 0.84-0.98), indicating that the ORPI had an excellent prognostic potency for this point. Setting the threshold at 0.2 offered the optimal compromise between specificity (86%) and sensitivity (89%) and between positive predictive value (96%) and negative predictive value (68%). At this cut-off level, the efficacy of the ORPI for collecting at least 4 oocytes was 88%. The ROC curves also revealed good prognostic potency for all other factors (Age, AMH and AFC) analysed. However, the AUC presented by the ORPI was always higher than those presented by all others factors. Figure 2A1 shows these data. Despite small differences, considering the ROC curves for GnRH-a and GnRH-ant groups individually showed that the ORPI also exhibited an excellent ability to predict collection of ≥4 oocytes (Figure 2A2 and 2A3).

Similarly, in regards to the probability of collecting ≥4 MII oocytes (Figure 2B), the ROC curve had an area under the curve of 0.84 (95% CI: 0.75-0.93), indicating that the ORPI also had a good prognostic potency for this issue. Setting the threshold at 0.3 offered the optimal compromise between specificity (73%) and sensitivity (85%) and between positive predictive value (86%) and negative predictive value (70%). At this cut-off level, the efficacy of the ORPI for retrieval of at least 4 MII oocytes was 81%. The ROC curves also revealed good prognostic potency for all other factors (Age, AMH and AFC) analysed. However, the AUC presented by the ORPI was always higher than those presented by all others. Again, the ROC curves demonstrate similar good results in the GnRH-a and GnRH-ant groups (Figure 2B2 and 2B3) in regards to the probability of collecting ≥4 MII oocytes.

In the same way, the ROC curve for the probability of collecting ≥15 oocytes (Figure 2C) gave an area under the curve of 0.89 (95% CI: 0.83-0.95), indicating that the ORPI values in this situation had a good prognostic potency. Setting the threshold at 0.9 offered the optimal compromise between specificity (89%) and sensitivity (86%) and between positive predictive value (96%) and negative predictive value (68%). At this cut-off level, the efficacy of the ORPI for collecting ≥15 oocytes was 82%. The ROC curves also revealed good prognostic potency for all other factors (Age, AMH and AFC) analysed. However, the AUC presented by the ORPI was always higher than those presented by all others. Regarding the probability of collecting ≥15 oocytes, the ROC curves for the GnRH-a and GnRH-ant groups indicated that the ORPI had a good prognostic potency for this point (Figure 2C2 and 2C3).

Finally, regarding the probability of pregnancy occurrence, the ROC curve showed an area under the curve of 0.74 (95% CI: 0.84-0.98), indicating that the ORPI had a good prognostic potency for this point. Setting the threshold at 0.3 offered the optimal compromise between specificity (47%) and sensitivity (93%) and between positive predictive value (46%) and negative predictive value (94%). At this cut-off level, the efficacy of the ORPI for pregnancy occurrence was 62%. ROC curves also revealed good prognostic potency for all other factors (Age, AMH and AFC) analysed. However, the AUC presented by the ORPI was always higher than those presented by all other factors. Figure 2D1 shows these data. Considering the ROC curves for GnRH-a and GnRH-ant groups individually, the ORPI also exhibited a good ability to predict clinical pregnancy (Figure 2D2 and 2D3).

## Discussion

A reliable indicator for supplying more precise estimates of the patients’ ovarian response might facilitate the optimisation and individualisation of assisted reproductive treatment before the onset of a treatment cycle. The present study proposes a new index, the ORPI, to identify the probable ovarian response to stimulation during the ART cycles. The combination of different variables in the ORPI resulted in a more precise index to predict the ovarian response. Indeed, the results showed significant correlations (*P*<0.001) between the ORPI values and the number of obtained follicles and the number and maturity of the collected oocytes. In addition, the results using the ORPI were always better than those results obtained using other predictive factors (AFC, AMH and age) separately. These findings support the use of this simple 3-variable index.

To the best of our knowledge, the present study is the first to combine those 3 factors into one single index for the assessment of the ovarian reserve. An estimate based solely on age is not always sufficient to accurately predict the ovarian response to gonadotropin stimulation, considering that the ovarian response is highly variable even among women of a similar age [5]. This inter-individual variation depends on the ovarian reserve of each person, which is influenced by genetic and environmental factors that primarily determine the size of the pool of primordial follicles at birth and the rate of the pool’s decline throughout the reproductive life [36,37]. In addition, the number of antral follicles can be assessed during a routine pelvic ultrasound examination, which is an integral part of the pretreatment assessment of women undergoing any assisted reproduction treatment in almost all fertility units. Therefore, an ultrasound evaluation of the antral follicles has gained acceptance as a good predictor of the ovarian response with low intra- and inter-observer variations [12,13], despite its routine use being hampered by the lack of a standard methodology that would enable valid data comparisons between different centres [6,38]. Based on these observations, a joint analysis of age and the AFC might combine their advantages and compensate for their disadvantages, thus improving the assessment of ovarian function. Indeed, upon attempting to develop prognostic models for the identification of patients’ ovarian response, la Cour Freiesleben et al. [39] found that the best prognostic model to predict a low response included AFC and age.

In addition, the prediction of the ovarian response could be further improved by including the serum AMH levels into the calculation of the ORPI. Despite this test not being universally available and recent alterations in the methodology [14-16], the determination of the AMH level consists of a simple blood test that can be performed at any time during the menstrual cycle [40,41]. In contrast to the levels of FSH, LH and oestradiol, the levels of AMH throughout the menstrual cycle show no consistent fluctuation patterns [42]. Moreover, the random fluctuations were small, indicating that AMH can be used as a reliable and cycle-independent marker for the ovarian reserve [16,43-45]. AMH appears to have a strong association with the ovarian response to stimulation, as shown by several authors [10,18,19,46-50], and it was eventually suggested for use in individualising the regimens for ovulation stimulation based on the AMH values [31-33]. In 2 meta-analyses, Broe et al. [34,35] found that AMH exhibits the same level of precision as the AFC for predicting poor ovarian response and excessive responders to ovarian stimulation. Jayaprakasan et al. [10] emphasised that AMH and AFC might replace each other as the best predictors of a poor ovarian response.

The simplicity of the calculation, which requires clinicians to perform simple mathematical operations using the variable values directly, and a direct correlation with the results are the major strong points of the ORPI. Other published studies also aimed to assess the ovarian response by combining variables [9,11,20,22,39]. However, either these formulas were too complex compared to the simplicity of the ORPI [9,24] or a wide variety of variables were included, which made the assessment complex [11,20,22]. Other studies [51,52] described an index whose calculation required at least 1 cycle of treatment. Conversely, another advantage of the ORPI is its ability to estimate the ovarian response before the onset of any treatment.

The present study has potential limitations. First, despite recruiting all eligible participants during the study period, the sample size is limited. This point is particularly important in regards to how the outcomes relate to clinical pregnancy. Despite the good results observed, the relationship will strengthen with analysis of larger numbers of cycles/patients. Second, FSH dose adjustments after the first ultrasound did not follow a strict protocol. Third, the study is not randomized trail and there is a possibility of bias due to the difference in the sample size between GnRH agonist and GnRH antagonist group. However, it should be noted that all analyzes showed no differences between the two protocols. Finally, despite basing our response definitions on numbers found in published studies, different reproduction clinics may prefer other definitions for low/excessive ovarian responses, which could modify the cut-offs indicated. These limitations indicate that the ORPI thresholds observed in our study could be prospectively further re-evaluated. However, considering the good results that our study showed, the thresholds of ORPI can be used for counselling clinicians regarding the realistic chances of pregnancy for patients.

There is no conventional ovarian stimulation regimen universally useful for every single patient. Based on its predictive potential, the ORPI might be used as a tool in the individualised planning of the medication doses and/or ovarian stimulation regimens. Based on the cut-off points obtained by the ROC curve analysis, we suggest several stimulation regimens grounded on the results of the ORPI. Table 3 summarises these stimulation protocols and the FSH doses to be used according to the range of calculated ORPI values with a particular focus on the extreme points of the ovarian response. ORPI values of <0.2 were shown to have the best sensitivity (86%) and specificity (89%) in predicting a poor ovarian responder. Similarly, an ORPI of ≥0.9 was shown to have the best sensitivity (89%) and specificity (86%) in predicting a high ovarian responder. In these cases, the use of protocols including GnRH antagonists and regimens with low-dose gonadotropin were recommended to reduce the ovarian response and minimise the occurrence and/or severity of ovarian hyperstimulation syndrome [53].

**Table 3:** The deployment of the ovarian stimulation protocol and doses of follicle-stimulating hormone (FSH) in the groups categorised by the ovarian response prediction index (ORPI)

**ORPI values**	**Oocyte number (expected)**	**Protocol**	**Dose of FSH**
<0.2	≤3	-GnRH Antagonist -Short GnRH Agonist -Clomiphene citrate + FSH -Long GnRH Agonist	300 IU-150 IU
≥0.2-<0.5	4-5	-GnRH Antagonist -Short GnRH Agonist -Long GnRH Agonist	300 IU-150 IU
≥0.5-<0.9	6-14	-Long GnRH Agonist -GnRH Antagonist	150 IU-112.5 IU
≥0.9	≥15	-GnRH Antagonist	112.5 IU-75 IU

## Conclusions

To summarise, the present study describes a new ORPI, which is a simple 3-variable index that exhibits an excellent ability to predict a low ovarian response (AUC: 0.91) and a good ability to predict the collection of >4 MII oocytes (AUC: 0.84) and an excessive ovarian response (AUC: 0.89) in infertile women. The ORPI might be used to improve the cost-benefit ratio of ovarian stimulation regimens by guiding the selection of medications and by tailoring the doses and regimens to the actual needs of patients.

## Competing interests

The authors declare that they have no competing interests.

## Authors’ contributions

JBAO participated in the design of the study, data interpretation, and the drafting of the manuscript. All authors were responsible for the data collection, analysis, and interpretation presented in the manuscript. RLRB participated in the design of the study, data collection, and the coordination of the study. JGFJ determined the variables involved in calculation of the ORPI and participated in critical revision of the manuscript. All authors read and approved the final manuscript.
